# Flooding and Cognitive Health among Middle-Aged and Older Adults in Thailand: A Case Study of Resilient City Policy in Bangkok

**DOI:** 10.5334/aogh.4740

**Published:** 2025-08-19

**Authors:** Fei Sun, Jin Ke, Phatchanun Vivarakanon, Myo Nyein Aung, Qing Xia, Leiwen Jiang

**Affiliations:** 1 School of Social Work, Michigan State University, East Lansing, USA; 2 Aging Service Research Center, School of Sociology, Huazhong University of Science and Technology, Wuhan, China; 3 Boromarajonani College of Nursing Nakhon Lampang, Faculty of Nursing, Praboromarajchanok Institute, Thailand; 4 Department of Global Health Research, Graduate School of Medicine, Juntendo University, Tokyo, Japan; 5 The Institute for Global Health, Michigan State University, East Lansing, USA; 6 Asian Demographic Research Institute, Shanghai University, Shanghai, China

**Keywords:** flooding and cognitive health, middle-aged and older adults, resilient city program, Thailand

## Abstract

*Background:* Thailand, a developing country in Southeast Asia, faces significant challenges due to urbanization, population aging, and climate change. This case study focuses on an adaptation strategy implemented in Bangkok to address the impacts of flooding. The study aims to examine the short-, medium-, and long-term effects of flooding exposure on cognitive health among middle-aged and older adults in Thailand and to identify the role of resilient city policy (RCP) in mitigating these impacts.

*Methods:* Data on cognitive health (memory, calculation, and orientation to time) were obtained from the three waves (2017, 2020, and 2022) of the Health, Aging, and Retirement (HART) surveys. Flooding data were retrieved from Thai flood hazard records in the EM-DAT database from 2017 to 2022. Panel data analyses with a fixed effects model were used to estimate the effects of flooding on cognitive health and the moderating effects of RCP.

*Results:* Findings indicate that exposure to flooding negatively impacts memory and orientation to time, with these effects worsening over time. RCP was found to mitigate the negative effects of flooding on memory scores and calculation scores. Additionally, RCP was associated with reduced risks for diabetes and depressive symptoms, indirectly protecting cognitive health.

*Discussion:* Flooding has long-lasting negative effects on certain cognitive health domains, specifically memory and orientation to time. This study suggests that RCP, a multi-component policy aimed at improving structural systems, community preparedness, and healthcare access, shows promise in mitigating the adverse effects of flooding on residents’ cognitive health. Further research is needed to link specific policy components to cognitive health outcomes and to understand their roles in protecting cognitive health.

## 1. Background

Temperature increases and extreme weather events due to climate change are predicted to enhance the frequency and severity of flooding globally [1], and particularly in Southeast Asia [2]. Thailand, a developing country in Southeast Asia, faces significant flooding challenges due to its geographical location. Systematic evidence suggests the flooding and related secondary stressors contributed to high rates of post-traumatic stress disorder (PTSD), depression, and anxiety among affected populations [3]. Older adults are at heightened risk due to limited literacy about disaster preparation, existing health conditions, and financial constraints, all of which negatively affect their physiological and psychological health [4]. Thailand’s demographic structure highlights a crucial need to examine the vulnerability of its aging population to natural disasters. Studying the impact of climate change-related disasters and related adaptation policy strategies on this population is therefore needed.

### 1.1 Climate stressor: flooding in Thailand

Bangkok, the capital city of Thailand, is in flood-prone areas facing severe flooding threats. Climate change, in particular, the rising dew point temperature, has been found to contribute to the increased frequency of flooding in Bangkok [5]. Urban environmental problems, such as high population density and poor infrastructure, also exacerbated flooding risks. Millions of Bangkok residents suffered from the 2011 flood, the most severe one in Thailand’s history, which resulted in considerable fatalities and economic losses. In subsequent years until recently, Bangkok has continued to experience monsoon floods and flash floods due to heavy rainfall and storms [6].

### 1.2 The influence of flooding on cognitive health

Studies of the health impact of flooding have largely focused on physical and psychological health [7], but flooding may also affect an individual’s cognitive health [3], referred to as a range of brain abilities, such as memory, orientation to time and space, thinking, learning, reasoning, and decision-making. The consequences can be substantial for middle-aged and older adults, who face age-related cognitive risks.

Theoretically, flooding can directly and indirectly affect the cognitive health of middle-aged and older individuals. From a neuropsychological perspective, the acute stress due to flooding significantly impairs an individual’s cognitive health, such as attention, working memory, and verbal memory [8, 9]. From a brain science perspective, acute and chronic stress caused by flooding induces microstructural changes in the prefrontal region of the human brain, with significant decreases in white matter density in the cingulum, both in the short- and long-term post-disaster [10, 11]. Persistently low white matter density in the cingulum indicates the presence of cognitive dysfunction [12].

### 1.3 Adaptation strategy: resilient city policy (RCP) in Bangkok

RCP aimed to build urban resilience by addressing issues such as climate change, natural disasters, and socio-economic disparities [13]. It has three strategic areas: (1) improving quality of life, (2) reducing risks and improving adaptation, and (3) driving a strong and competitive economy. The first two strategies are of crucial importance to addressing the flooding effects on the cognitive health of residents examined in this study.

At the city level, the RCP can generate direct benefits by improving urban flood defenses, such as road drainage systems, drainage tunnels, and major canals. These measures can reduce flooding incidence and severity, enhancing the living environment and directly improving cognitive health [14]. At the community level, RCP initiatives supported community disaster risk pilots and a network of community leaders who build capacity for disaster and climate risk management, mobilize resources, and improve flood communication. Enhanced community outreach, social engagement, and support from these initiatives may contribute to cognitive health [15].

Specifically, this study aimed to address the following three research questions: (1) What are the short-, medium-, and long-term effects of flooding on the cognitive health of middle-aged and older adults in Thailand; (2) How may the RCP directly mitigate the effect of flooding on cognitive health; and (3) How the RCP may indirectly mitigate the effect of flooding through reduced health and mental health risks (i.e., depression, diabetes, and sleep).

## 2. Approach

This case study mainly used two secondary data sources: the* Health, Aging, and Retirement in Thailand (HART) surveys* and Thai flood hazard records from the EM-DAT database.

### 2.1 Data

#### 2.1.1 HART surveys

Data on the cognitive health status of middle-aged and older adults in Thailand were obtained from the HART survey conducted in 2017, 2020, and 2022. Participants in the HART surveys provided written informed consent, and the study was approved by the Ethics Committee in Human Research of the National Institute of Development Administration ECNIDA (ECNIDA 2020/00012).

HART had a sample size of about 5,600 individuals from five regions of Thailand and collected information on demographic characteristics, health and cognition, employment, income, retirement, assets, liabilities, life expectancy, and satisfaction of the sample [16]. In 2015, the HART released the first wave of data, with cognitive health-related data available in 2017 (Wave 2), 2020 (Wave 3), and 2022 (Wave 4) [17]. A detailed list of 13 provinces, including Bangkok and surrounding areas in the HART sample, can be found in Appendix A.

#### 2.1.2 Flood data

The database includes disasters meeting at least one of the following criteria: a minimum of 10 deaths, 100 people or more affected, declaration of a state emergency, or provision of international assistance. EM-DAT records detailed information on disaster type, starting and ending dates, and geographic information (including latitude, longitude, and the administrative area) [18–20].

**Figure 1:**
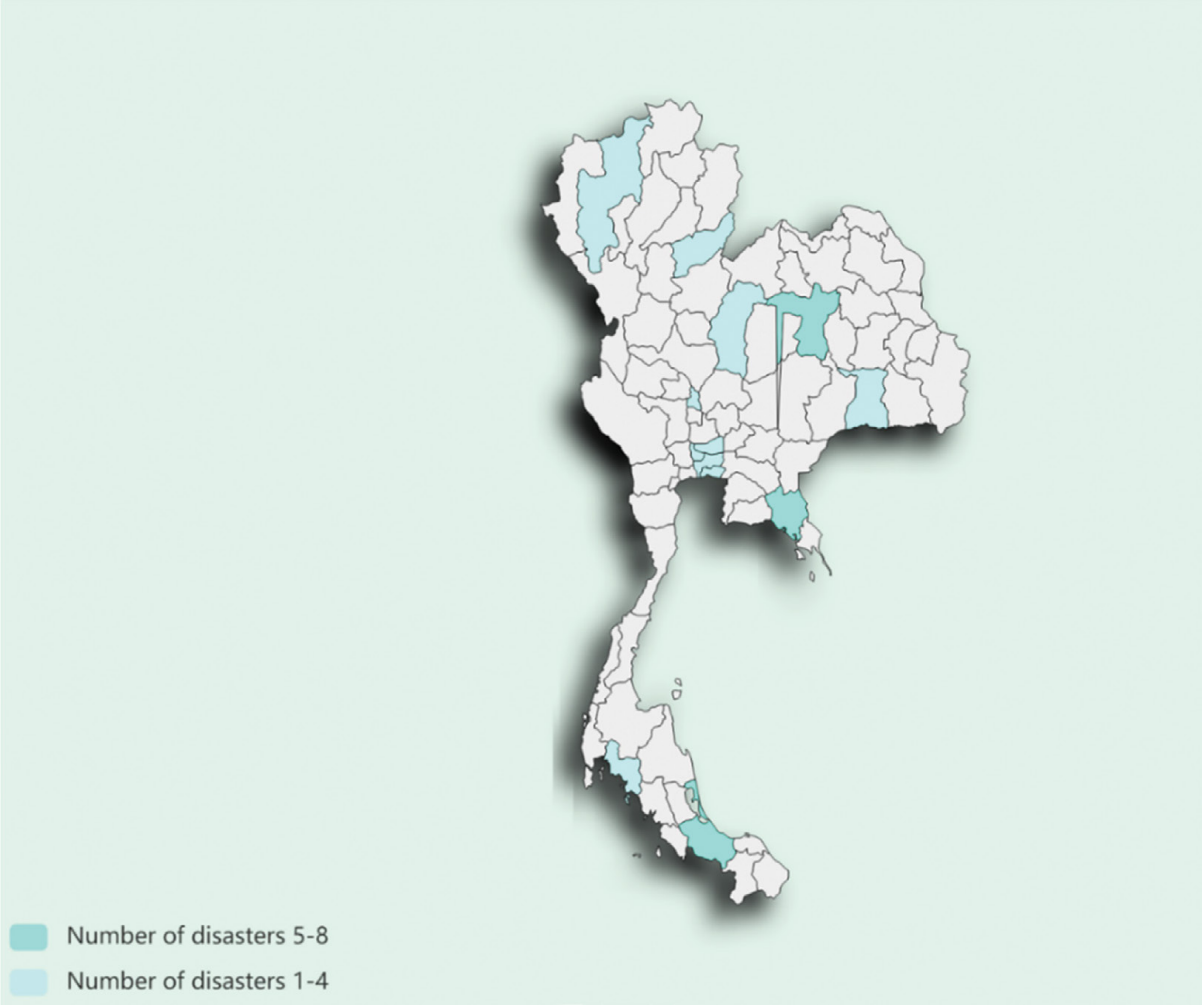
Distribution of floods in Thailand, 2017–2022. *Note*: Khon Kaen (40) and Songkhla (90) are two provinces that experienced disasters with frequencies between 5 and 8, while all other provinces experienced frequencies of 4 or below. Details can be found in Appendix B.

This study used Thai flood hazard records from the EM-DAT database for the period 2017–2022, covering 13 provinces in the HART survey. The selected period aligned with three waves of HART data from 2017 to 2022. Figure 1 shows the distribution of the 2017–2022 floods in Thailand included for consideration, as detailed in the list in Appendix B. We merged Thai flood record data with HART data at the provincial level across all HART waves.

#### 2.1.3 Measures in analyses

**Flooding exposure** was captured in the short, medium, and long term, and with reference to the strategy of related studies [21, 22]. The flooding exposure variables in this paper are defined as follows: (1) Short-term exposure was defined as the experience of flooding that occurred up to one year prior to the individual interview date. (2) Medium-term exposure was defined as experience of flooding that occurred between one and three years prior to the individual interview date. (3) Long-term exposure was defined as an experience of flooding that occurred more than three years prior to the individual interview date. All three exposure variables were coded as dummy variables to avoid double counting of floods that may have occurred over multiple months. Each variable was assigned a value of 1 if the individual was exposed to flooding during the corresponding period prior to the interview date, and 0 otherwise.

**RCP access.** Samples residing in the Bangkok metropolitan area and with observation waves in 2020 and 2022 were coded as 1, indicating that the RCP was available, and the other samples were coded as 0. This is determined by the specific planning and implementation timeframe of the RCP, and while the RCP has been in place since 2017, key measures were not completed in 2017 or have not yet been implemented [23].

**Cognitive health** in the HART was assessed along three domains: (1) memory measured by a word recall test using immediate and delayed recall tests, each containing 10 words, totaling 20 points; (2) calculation measured by a series of deductions of seven from 100, totaling five points; and (3) orientation to time measured by awareness of time, including year, month, day, and weekday of the interview date, totaling four points [24]. Given the lack of a global cognitive health measure including other cognitive domains such as judgment, attention, and executive function, creating an aggregated score for overall cognitive health was not considered appropriate. Instead, our analyses focused on each of the three individual cognitive domains separately.

Three mediating mechanism variables were obtained from the HART survey. Depression was measured using the 10-item version of the Center for Epidemiologic Studies Depression Scale (CES-D-10). When an individual’s CES-D-10 score was greater than or equal to 10, the individual was considered to have depressive symptoms and assigned a value of 1, and otherwise was assigned a value of 0 [25]. Diabetic condition was measured by severity rating (1 indicating becoming more severe, 0 indicating otherwise) [26], and sleep duration was measured by hours of sleep.

Covariates for cognitive health included individual- and provincial-level factors. Individual-level covariates covered demographics, health behaviors, and household aspects, including age, gender, marital status, years of education, employment status, religion, whether or not they lived alone, urban or rural residence, whether or not they smoke, whether or not they drink, whether or not they exercise, whether or not they have annual medical examination and family assets [27–29]. Furthermore, to account for regional environmental and socioeconomic factors that may confound the relationship between flooding and cognitive health, we controlled for two provincial-level covariates [30, 31]. First, provincial GDP per capita was included as a key indicator of regional socioeconomic status. This variable, obtained from the National Economic and Social Development Office of Thailand, serves as a proxy for access to resources, quality of public services, and overall healthcare infrastructure, all of which are known to influence cognitive outcomes. Second, vegetation cover at the provincial level was controlled using the Normalized Difference Vegetation Index (NDVI), with data obtained from the Food and Agriculture Organization of the United Nations. NDVI is a satellite-derived index that measures the density of green vegetation. We included this covariate because a growing body of research indicates that greater exposure to green space is associated with reduced stress and depression and better cognitive function. Controlling NDVI allowed us to isolate the direct impact of flooding from the background influence of the natural environment on cognitive health.

#### 2.1.4 Panel data characteristics

A final unbalanced panel dataset containing 10,058 observations (2,168 in 2017, 3,088 in 2020, and 4,802 in 2022) was created using a list-wise deletion method (see Table 1 for descriptive statistics by year). The average age of the sample was 65.9 (SD = 11.3) in 2017, with slightly more than half being female, about half living in rural areas, one-fourth living alone, and more than 90% of the sample being Buddhists. Descriptive statistics of cognitive test scores are detailed in Appendix C.

**Table 1:** Descriptive statistics of participants.

	2017 WAVE (*N* = 2,168)			2020 WAVE (*N* = 3,088)			2022 WAVE (*N* = 4,802)		
	MEAN	SD	*N*	MEAN	SD	*N*	MEAN	SD	*N*
Age	65.9100	11.3297	2168	69.4090	11.3482	3088	66.7791	11.4521	4802
Gender (Male = 1; Female = 0)	0.4515	0.4978	2168	0.4061	0.4912	3088	0.3813	0.4858	4802
Married (Yes = 1; No = 0)	0.6752	0.4684	2168	0.5852	0.4928	3088	0.5796	0.4937	4802
Working (Yes = 1; No = 0)	0.3904	0.4880	2168	0.3277	0.4695	3088	0.4640	0.4988	4802
Years of education	6.8080	2.8391	2168	6.5855	2.7680	3088	7.2422	3.0787	4802
Religion									
No religion (Yes = 1; No = 0)	0.0000	0.0000	2168	0.0006	0.0254	3088	0.0002	0.0144	4802
Buddhist (Yes = 1; No = 0)	0.9180	0.2741	2168	0.9129	0.2820	3088	0.9263	0.2613	4802
Christian (Yes = 1; No = 0)	0.0020	0.0479	2168	0.0039	0.0622	3088	0.0050	0.0705	4802
Muslims (Yes = 1; No = 0)	0.0790	0.2705	2168	0.0823	0.2748	3088	0.0660	0.2483	4802
Other (Yes = 1; No = 0)	0.0000	0.0000	2168	0.0003	0.0180	3088	0.0025	0.0499	4802
Living alone (Yes = 1; No = 0)	0.2384	0.4262	2168	0.1308	0.3373	3088	0.1143	0.3182	4802
Urban residents (Yes = 1; No = 0)	0.4851	0.4999	2168	0.4812	0.4997	3088	0.5702	0.4951	4802
Smoking (Yes = 1; No = 0)	0.1410	0.3481	2168	0.1069	0.3090	3088	0.1056	0.3073	4802
Drinking (Yes = 1; No = 0)	0.2030	0.4023	2168	0.1253	0.3311	3088	0.1808	0.3849	4802
Exercise (Yes = 1; No = 0)	0.5526	0.4973	2168	0.5097	0.5000	3088	0.5092	0.5000	4802
Medical examination (Yes = 1; No = 0)	0.5613	0.4963	2168	0.5946	0.4911	3088	0.4536	0.4979	4802
Logarithm of family assets	12.7929	1.6424	2168	7.2202	6.3240	3088	4.1983	5.9692	4802
Logarithm of GDP per capita	11.8638	0.4507	2168	11.9013	0.5056	3088	11.9950	0.5409	4802
Vegetation cover (NVDI)	0.6409	0.0900	2168	0.5862	0.0925	3088	0.6188	0.0079	4802

Note: SD = standard deviation. GDP = gross domestic product. NVDI = normalized difference vegetation index.

### 2.2 Analysis strategies

The basic econometric model of this paper is as follows:


Scoreijt=α1Ti,t−365+α2Ti,t−365_t−1095+α3Ti,t−1095_t−∞+δX′ijt+ E2+E3+λ+iχj+ηt+εijt 1

where ${Score}_{ijt}$ was the cognitive score of individual i in province j at date t. $T_{i,t-365}$ measured an individual’s exposure to flooding in the year prior to date t, i.e., short-term exposure. $T_{i,t-365\_t-1095}$ measured an individual’s exposure to flooding between one and three years prior to date t, i.e., medium-term exposure. $T_{i,t-1095\_t-\infty }$ measured an individual’s exposure to flooding more than three years prior to date t, i.e., long-term exposure. $X_{ijt}$ denoted a set of covariates. $E_{2}$ and $E_{3}$ indicated whether individuals were exposed to flooding during any two or all three periods considered, respectively. This was to exclude the potential effect of individuals experiencing multiple floods over time on the estimates in this paper. $\lambda_{i}$ denoted individual fixed effects. $\chi_{j}$ represented the provincial-level fixed effect, which could not be absorbed by the individual fixed effect because some individuals did not reside in the same province across waves. $\eta_{t}$ indicated year, interview month, and day fixed effects. $\varepsilon_{ijt}$ was the error term, and the standard errors were clustered at the provincial level.

Furthermore, to examine the effects of the RCP, we adjusted model (1) to the following form:


$$\begin{array}{ccc} Y_{ijt} & = & \alpha_{1}T_{i,t-365}\times RCP+\alpha_{2}T_{i,t-365\_t-1095}\times RCP+\alpha_{3}T_{i,t-1095\_t-\infty }\times RCP\\ & & +\;\alpha_{4}T_{i,t-365}+\alpha_{5}T_{i,t-365\_t-1095}+\alpha_{6}T_{i,t-1095\_t-\infty }+\alpha_{7}RCP+\delta {X^{\prime }}_{ijt}\\ & & +\;E_{2}+E_{3}+\lambda_{i}+\chi_{j}+\eta_{t}+\varepsilon_{ijt} \end{array}$$ 2

$Y_{ijt}$ represented cognitive test scores and other channeled variables of the flood that affect cognitive health. RCP indicated whether an individual was covered by the *RCP*. By introducing the interaction terms of RCP and $T_{i,t-365}$, $T_{i,t-365\_t-1095}$, and $T_{i,t-1095\_t-\infty }$ in model (1), as well as the main effect term of RCP, we were able to distinguish the short-, medium- and long-term effects of the RCP.

## 3. Findings

### 3.1 The effect of flooding

**Figure 2:**
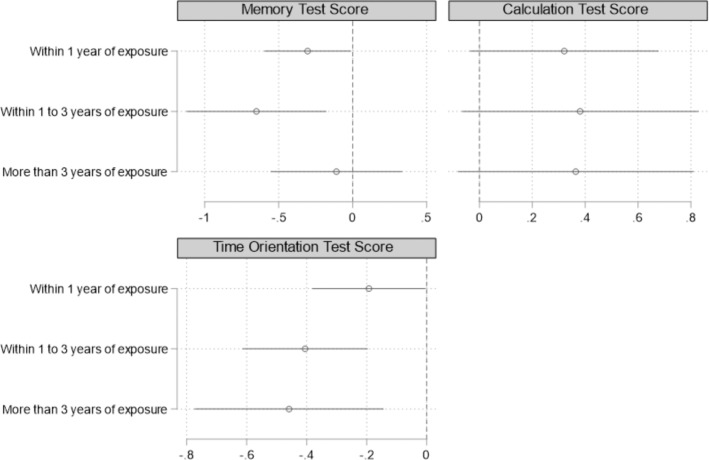
The effect of flooding on cognitive health among middle-aged and older residents in Thailand. *Note*: 90% confidence intervals were reported in the figure. All regression models were conducted in the same form as model (1). This figure reports standardized coefficients. Specific regression results were detailed in Appendix D. The standard errors clustered at the provincial level were in parentheses.

Figure 2 reports the estimates of the effect of flooding on cognitive test scores across exposure periods (see detailed regression results in Appendix D). First, flooding significantly negatively affected memory and orientation to time, both within one year and between one and three years since flooding. More than three years since the flood, the fact that the flooding effect was still significant regarding orientation to time. This implies that flooding was detrimental to individual memory and orientation to time and that this negative effect on orientation to time particularly persisted over time.

Second, the negative effects of flooding tended to exacerbate over time. Within one year of the disaster, flooding decreased scores on the memory test by 0.305 SD and scores on the time orientation test by 0.193 SD, while between one and three years after the disaster, flooding decreased scores on the memory test by 0.651 SD and scores on the time orientation test by 0.405 SD. More than three years after the disaster, flooding decreased scores on the date and time test by 0.459 SD.

### 3.2 The effects of resilient city policy

#### 3.2.1 Direct effects of resilient city policy

**Figure 3:**
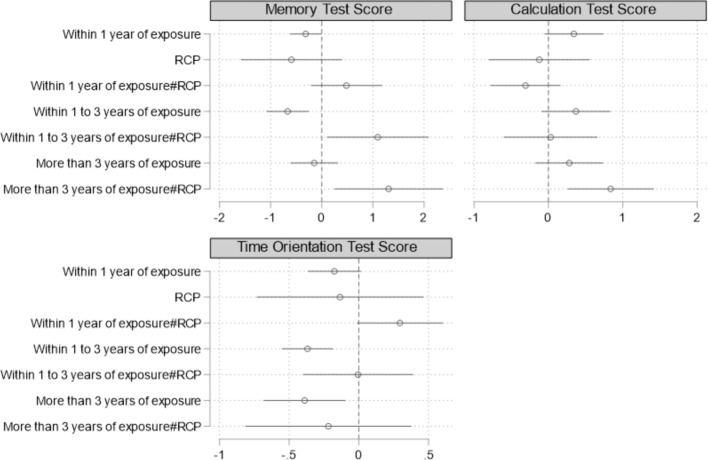
The effect of the Bangkok RCP on cognitive health. *Note*: 90% confidence intervals were reported in the figure. Specific regression results were detailed in Appendix E1. This figure reports standardized coefficients. All regression models were conducted in the same form as model (2). RCP indicates resilient city policy. The standard errors clustered at the provincial level were in parentheses.

Figure 3 reports the direct intervention effects of the RCP. Specific regression results are detailed in Appendix E1. Between one and three years post-disaster, the RCP intervention increased the memory test scores of the affected individuals by 1.095 SD, and more than three years after the disaster, the RCP intervention increased the memory test scores by 1.307 SD, and the effect of the intervention gradually increased with time. Furthermore, although flooding did not have a significant effect on calculation test scores, the RCP intervention increased the calculation test scores of affected individuals by 0.837 SD more than three years after the disaster. These results imply that the RCP could be promising in protecting certain cognitive domains, such as memory and calculation, but might not be for other cognitive domains such as orientation to time.

#### 3.2.2 Possible indirect effects of resilient city policy

**Figure 4:**
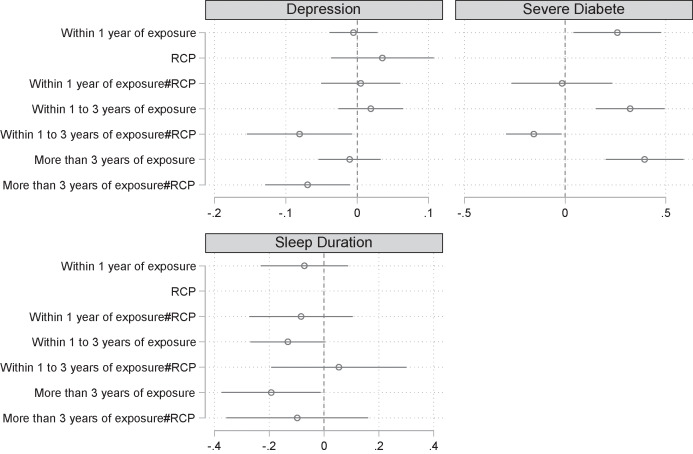
The effects of the Bangkok RCP on risk factors for cognitive health. *Note*: 90% confidence intervals were reported in the figure. See Appendix E2 for specific regression results. All regression models were set up in the same form as model (2). RCP indicates resilient city policy. Missing results are omitted because of collinearity. The standard errors clustered at the provincial level are in parentheses.

The results imply that RCP could have promising effects in mitigating the impact of flooding on cognitive health via health or mental health factors (See Figure 4). See Appendix E2 for specific regression results.

First, RCP reduced the risk of depression among affected individuals by 8.1% between one and three years after the disaster and by 7% more than three years after the disaster. This finding should be contextualized by the fact that the main effect of flooding on the risk of depression was not significant. For risks of diabetes severity, flooding increased the risk of exacerbation in individuals with pre-existing diabetes by 25.9% within one year of the disaster, 32.3% within one to three years of the disaster, and 39.5% more than three years after the disaster, respectively, and this negative effect increased progressively with time. The RCP intervention reduced the risk of exacerbation in individuals with existing diabetes by 15.5% within one to three years of the disaster. Regarding sleep duration, flooding reduced sleep duration by 0.193 h in affected individuals more than three years after the disaster. But RCP did not have a significant effect of flooding on sleep duration.

### 3.3 Robustness tests

Robustness checks were performed. First, our identification assumption relied on the fact that the occurrence of floods was unpredictable for individuals, and then some time-varying, unobserved factors did not affect estimates. To test whether this assumption holds, we designed a falsification test [22, 32] to examine whether floods occurring one year or more after the interview affected test scores. The results showed that floods occurring one year or more after the interview do not affect test scores, which largely eliminates concerns about potential omitted variables, as detailed in Appendix F1. Second, to test for the effect of potential migration that may have occurred during the sample period, we constructed a dataset that retained only the sample that did not migrate in any of the three waves of the survey and re-estimated it. The results remain robust and are detailed in Appendix F2. Finally, we were worried that our results would be driven by certain severe floods, so we excluded floods that affected more than 1 million people and then re-estimated. The results remain robust, as detailed in Appendix F3.

## 4. Discussion

Our study confirmed the negative impact of flooding exposure on cognitive health, extending the existing literature that primarily examined flooding’s effects on psychological stress and physical health [33, 34]. We found that flooding exposure negatively affected cognitive health, including memory and orientation to time, both in the short and long term. Word recall and orientation to time are crucial indicators of cognitive health. Cognitive decline often begins with short-term memory issues, such as forgetfulness and difficulties in storing and retrieving new information. The observed significant impact of flooding on cognitive health occurred within the first year of the disaster and appeared to exacerbate during the intermediate period (i.e., one to three years). This finding aligns with literature suggesting more salient delayed health effects of flooding over time, because during the rebuilding phase after a disaster, secondary stressors, such as economic pressures, may emerge and further impact residents’ psychological well-being [35]. These findings affirmed the need for public policies like RCP to address these health impacts.

The RCP represents a shift from “fighting flooding” to “living with water” [23]. This approach not only emphasizes recovery from flooding but also promotes a comprehensive strategy to adapt to changing socio-ecological conditions. The two main components of the RCP target (1) improving the quality of life of the population by enhancing access to healthcare and a healthy lifestyle and (2) building community capacity to plan for foreseeable risks and adapt to flood hazards through infrastructure improvements (e.g., drainage systems, water management) and community organization and preparedness. Such measures addressed physical, psychological, social and environmental risks to cognitive health by improving healthcare access, promoting a healthy lifestyle, reducing emotional stressors, and improving a safe, inclusive physical environment. Although it is difficult to pinpoint the role of specific measures, the evidence of improved memory suggests promising effects of these policy initiatives.

Our findings partially supported the indirect effects of the RCP by moderating the effects of flooding on depression, diabetes, and sleep duration, and subsequently protecting cognitive health. Initially, we observed that exposure to flooding contributed to increased severity of diabetes and a reduction in sleep duration for individuals. These findings aligned with previous research that highlights the adverse health impacts of flooding [34]. RCP was found to mitigate these negative effects of flooding on diabetes. Similarly, we found that RCP contributed to a reduction in depression among participants. Through its comprehensive strategy that included enhanced healthcare access and safe environments, RCP may have helped reduce the health burden that may be exacerbated by flooding. For instance, better access to healthcare can lead to more effective management of diabetes. Consequently, by addressing these social drivers of health, RCP indirectly supports better cognitive health outcomes. Therefore, our findings provided evidence suggesting that RCP not only aided in direct flood recovery but also played a role in alleviating the secondary health impacts of flooding, thus contributing to the overall cognitive well-being of individuals in flood-prone areas. However, further research is needed to examine its effects on other cognitive domains and the underlying mechanisms.

The differential effects of the RCP on various cognitive domains were unexpected. RCP was found to contribute to improved short-term recall memory and calculation abilities in the long term, but it had no effect on orientation to time and day. Both short-term recall memory and calculation involve more complex cognitive processes, such as attention, encoding, storage, retrieval, and reasoning. These abilities are more relevant to daily problem-solving than orientation to time, making them more likely to be targeted in cognitive stimulus interventions. It is highly possible that the activities within the RCP framework provided cognitive stimulation through health education and community participation. However, orientation to time may receive less emphasis due to its lower importance and the ease with which older retirees can compensate for it (e.g., checking a calendar).

This case study shed light on the impact of flooding on cognitive health and alluded to the promising evidence of RCP in reducing such negative flooding impact.

The RCP has three major components, including one not elaborated in this study, which is economic growth. To achieve sustainable growth in urban and rural areas, it is essential to create an environment that provides good employment opportunities [36]. The well-being and quality of life of urban residents depend strongly on the level of the urban environment, which operates within a complex network of interactions with cultural, economic, and social factors [37]. Future research is needed to examine the specific functions of each policy component in relation to cognitive health and uncover the roles of all possible underlying mechanisms (e.g., increased recreational and social activities).

This study has several limitations. First, the HART measures of cognitive health did not include a global cognitive health measure, limiting us to three separate cognitive domains. Second, we only explored certain underlying mechanisms between flooding and cognitive health due to the availability of measures in the HART survey. For example, variables related to physical and social activity engagement, critical to cognitive health, were not available. Lastly, as this study examined the RCP as a holistic adaptation strategy, more research is needed to investigate specific components of the RCP in relation to the impact of flooding on health. Additionally, this study calls for more research into how individual socioeconomic status and regional economic development might moderate the impact of flooding or the effects of RCP, as such impacts across socioeconomic groups and regions can be complex and present social justice considerations.

## 5. Conclusions

This study examines the impact of flooding on cognitive health among middle-aged and older adults in Thailand, focusing on the RCP. The findings highlight flooding’s long-term negative effects on memory and time orientation while demonstrating the RCP’s role in mitigating these impacts by enhancing cognitive function and reducing health risks like depression and diabetes. The research underscores the importance of integrating climate adaptation into urban policies, emphasizing infrastructure, environmental sustainability, and social inclusivity. Challenges included data integration complexities and limitations in cognitive health assessment. The study calls for interdisciplinary research, investment in capacity building, and policy innovation to address climate-related health risks. Future research should explore specific RCP components, conduct broader cognitive health assessments, and examine resilience determinants across different regions. Strengthening resilience policies through research and collaboration is crucial to protecting vulnerable populations from the growing threats of climate change.

## Data Availability

The data used in this study are publicly available. Analytical datasets generated during the study can be provided upon request.

## Additional Files

The additional files for this article can be found as follows:

Supplementary Appendix A. List of HART Survey Sample Source Changwats (Provinces).

Supplementary Appendix B. Thailand flood records for 2017–2022 used in this study.

Supplementary Appendix C. Descriptive statistics of cognitive test scores.

Supplementary Appendix D. The effect of flooding on cognitive test scores across exposure periods.

Supplementary Appendix E1. The effect of resilient city policy.

Supplementary Appendix E2. Indirect effects of resilient city policy.

Supplementary Appendix F1. Results of robustness tests.

Supplementary Appendix F2. Results excluding immigrant samples.

Supplementary Appendix F3. Results excluding heavy flooding.
