# Supplementary material for: Flooding and Cognitive Health among Middle-Aged and Older Adults in Thailand: A Case Study of Resilient City Policy in Bangkok
Source: Ann Glob Health. 2025 Aug 19;91(1):49. doi: 10.5334/aogh.4740 (PMC12372663; doi:10.5334/aogh.4740)
Supplement: Supplementary Appendix A. — List of HART Survey Sample Source Changwats (Provinces). [file agh-91-1-4740-s1.pdf]

Appendix A List of HART Survey Sample Source Changwats (Provinces)

HART Code	Changwat
10	Bangkok
11	Samut Prakan
12	Nonthaburi
13	Pathum Thani
17	Sing Buri
22	Chanthaburi
32	Surin
40	Khon Kaen
50	Chiang Mai
53	Uttaradit
67	Phetchabun
81	Krabi
90	Songkhla
