# Supplementary material for: Flooding and Cognitive Health among Middle-Aged and Older Adults in Thailand: A Case Study of Resilient City Policy in Bangkok
Source: Ann Glob Health. 2025 Aug 19;91(1):49. doi: 10.5334/aogh.4740 (PMC12372663; doi:10.5334/aogh.4740)
Supplement: Supplementary Appendix B. — Thailand flood records for 2017–2022 used in this study. [file agh-91-1-4740-s2.pdf]

Appendix B Thailand flood records for 2017-2022 used in this study

No.	Location (HART Code)	Start Year	Start Month	Start Day	End Year	End Month	End Day	Total Affected
1	81,91	2017	1	1	2017	1	31	1800000
2	22,67,53,50	2017	7	5	2017	8	2	1000000
3	10,13,17,40	2017	10	10	2017	11	8	605000
4	90	2017	11	25	2017	12	3	385498
5	40	2019	9	11	2019	9	11	158000
6	50,53	2020	8	22	2020	8	23	40000
7	90	2020	11	25	2020	12	14	691659
8	90	2021	1	5	2021	1	11	175493
9	22,17,11	2021	8	27	2021	8	31	325400
10	22,50,67	2021	9	19	2021	10	6	298900
11	22	2021	10	12	2021	10	15	25001
12	81,90	2021	11	29	2021	12	2	132900
13	53	2022	5	14	2022	5	18	1380
14	50	2022	5	20	2022	5	24	3900
15	10,40,11,12	2022	7	17	2022	7	26	2300
16	40	2022	8	14	2022	8	16	63100
17	40,13,67,17,32	2022	9	1	2022	10	6	485908
18	90	2022	11	8	2022	11	10	25605
19	90	2022	12	19	2022	12	21	630000

Notes: The specific start day of No. 17 flood is missing from the EM-DAT database, and we have filled in the start day as the 1st, based on the location of the disaster, using the published information on ReliefWeb, a humanitarian information platform created by the United Nations Office for the Coordination of Humanitarian Affairs (UN OCHA).
