# Supplementary material for: Flooding and Cognitive Health among Middle-Aged and Older Adults in Thailand: A Case Study of Resilient City Policy in Bangkok
Source: Ann Glob Health. 2025 Aug 19;91(1):49. doi: 10.5334/aogh.4740 (PMC12372663; doi:10.5334/aogh.4740)
Supplement: Supplementary Appendix C. — Descriptive statistics of cognitive test scores. [file agh-91-1-4740-s3.pdf]

Appendix C Descriptive statistics of cognitive test scores

	Non-exposed group<br>Mean (SD)	Exposure group<br>Mean (SD)	Total<br>Mean (SD)
Memory Test Score	6.040 (3.641)	7.787 (3.724)	7.400 (3.776)
Calculation Test Score	2.137 (1.727)	2.790 (1.853)	2.616 (1.843)
Time Orientation Test Score	3.304 (1.088)	3.777 (0.683)	3.695 (0.790)

Notes: SD = standard deviation.
