# Supplementary material for: Flooding and Cognitive Health among Middle-Aged and Older Adults in Thailand: A Case Study of Resilient City Policy in Bangkok
Source: Ann Glob Health. 2025 Aug 19;91(1):49. doi: 10.5334/aogh.4740 (PMC12372663; doi:10.5334/aogh.4740)
Supplement: Supplementary Appendix D. — The effect of flooding on cognitive test scores across exposure periods. [file agh-91-1-4740-s4.pdf]

Appendix D The effect of flooding on cognitive test scores across exposure periods

	Memory Test Score	Calculation Test Score	Time Orientation Test Score
Within 1 year of exposure	-0.362*	0.180	-0.108
	(0.184)	(0.190)	(0.122)
Within 1 to 3 years of exposure	-0.542***	0.302	-0.268**
	(0.148)	(0.259)	(0.108)
More than 3 years of exposure	0.094	0.338	-0.372**
	(0.286)	(0.202)	(0.179)
Individual FE	Y	Y	Y
Changwat (province) FE	Y	Y	Y
Year FE	Y	Y	Y
Interview month FE	Y	Y	Y
Interview day FE	Y	Y	Y
E_2 & E_3	Y	Y	Y
Covariates	Y	Y	Y
Observations	8015	5425	6616
R-squared	0.614	0.642	0.644

Notes: This table reports standardized coefficients. Standard errors clustered at the level of changwat (province) are in parentheses. FE indicates fixed effects. *** $p<.01$, ** $p<.05$, * $p<.1$
