# Supplementary material for: Flooding and Cognitive Health among Middle-Aged and Older Adults in Thailand: A Case Study of Resilient City Policy in Bangkok
Source: Ann Glob Health. 2025 Aug 19;91(1):49. doi: 10.5334/aogh.4740 (PMC12372663; doi:10.5334/aogh.4740)
Supplement: Supplementary Appendix E1. — The effect of resilient city policy. [file agh-91-1-4740-s5.pdf]

Appendix E The effect of resilient city policy

E1. Direct effects of resilient city policy

	Memory Test Score	Calculation Test Score	Time Orientation Test Score
Within 1 year of exposure	-0.315*	0.343	-0.175
	(0.170)	(0.220)	(0.106)
Within 1 year of exposure#RCP	0.481	-0.307	0.295
	(0.388)	(0.263)	(0.172)
Within 1 to 3 years of exposure	-0.667**	0.370	-0.368***
	(0.231)	(0.259)	(0.102)
Within 1 to 3 years of exposure#RCP	1.095*	0.030	-0.006
	(0.557)	(0.351)	(0.222)
More than 3 years of exposure	-0.147	0.280	-0.389**
	(0.258)	(0.254)	(0.163)
More than 3 years of exposure#RCP	1.307**	0.837**	-0.219
	(0.593)	(0.323)	(0.334)
RCP	-0.593	-0.122	-0.135
	(0.554)	(0.381)	(0.335)
Individual FE	Y	Y	Y
Changwat (province) FE	Y	Y	Y
Year FE	Y	Y	Y
Interview month FE	Y	Y	Y
Interview day FE	Y	Y	Y
E_2 & E_3	Y	Y	Y
Covariates	Y	Y	Y
Observations	8015	5425	6616
R-squared	0.619	0.645	0.645

Notes: This table reports standardized coefficients. Standard errors clustered at the level of changwat (province) are in parentheses. RCP indicates Resilient Cities Policy. FE indicates fixed effects. *** p<.01, ** p<.05, * p<.1
