# Supplementary material for: Flooding and Cognitive Health among Middle-Aged and Older Adults in Thailand: A Case Study of Resilient City Policy in Bangkok
Source: Ann Glob Health. 2025 Aug 19;91(1):49. doi: 10.5334/aogh.4740 (PMC12372663; doi:10.5334/aogh.4740)
Supplement: Supplementary Appendix E2. — Indirect effects of resilient city policy. [file agh-91-1-4740-s6.pdf]

E2. Indirect effects of resilient city policy

	Depression	Severe Diabete	Sleep Duration
Within 1 year of exposure	-0.005	0.259*	-.072
	(0.019)	(0.122)	(0.090)
Within 1 year of exposure#RCP	0.005	-0.015	-0.084
	(0.031)	(0.140)	(0.106)
Within 1 to 3 years of exposure	0.019	0.323***	-0.132
	(0.025)	(0.096)	(0.077)
Within 1 to 3 years of exposure#RCP	-0.081*	-0.155*	0.054
	(0.041)	(0.078)	(0.139)
More than 3 years of exposure	-0.011	0.395***	-0.193*
	(0.024)	(0.108)	(0.101)
More than 3 years of exposure#RCP	-0.070*	——	-0.098
	(0.033)	——	(0.145)
RCP	.036	——	——
	(0.040)	——	——
Individual FE	Y	Y	Y
Changwat (province) FE	Y	Y	Y
Year FE	Y	Y	Y
Interview month FE	Y	Y	Y
Interview day FE	Y	Y	Y
E_2 & E_3	Y	Y	Y
Observations	10051	1447	7890
R-squared	0.539	0.806	0.623

Notes: Standard errors clustered at the level of changwat (province) are in parentheses. RCP indicates Resilient City Policy. Missing results are omitted because of colinearity. FE indicates fixed effects. *** p<.01, ** p<.05, * p<.1
