# Supplementary material for: Flooding and Cognitive Health among Middle-Aged and Older Adults in Thailand: A Case Study of Resilient City Policy in Bangkok
Source: Ann Glob Health. 2025 Aug 19;91(1):49. doi: 10.5334/aogh.4740 (PMC12372663; doi:10.5334/aogh.4740)
Supplement: Supplementary Appendix F1. — Results of robustness tests. [file agh-91-1-4740-s7.pdf]

Appendix F. Results of robustness tests

F1. Falsification test

	Memory Test Score	Calculation Test Score	Time Orientation Test Score
floods occurring 1 year and more after the interview	-0.046	0.343	0.055
	(0.195)	(0.265)	(0.132)
Individual FE	Y	Y	Y
Changwat (province) FE	Y	Y	Y
Year FE	Y	Y	Y
Interview month FE	Y	Y	Y
Interview day FE	Y	Y	Y
E_2 & E_3	Y	Y	Y
Covariates	Y	Y	Y
Observations	8015	5425	6616
R-squared	0.592	0.639	0.640

Notes: Standard errors clustered at the level of changwat (province) are in parentheses. This table reports standardized coefficients. FE indicates fixed effects. *** p<.01, ** p<.05, * p<.1
