# Supplementary material for: Flooding and Cognitive Health among Middle-Aged and Older Adults in Thailand: A Case Study of Resilient City Policy in Bangkok
Source: Ann Glob Health. 2025 Aug 19;91(1):49. doi: 10.5334/aogh.4740 (PMC12372663; doi:10.5334/aogh.4740)
Supplement: Supplementary Appendix F2. — Results excluding immigrant samples. [file agh-91-1-4740-s8.pdf]

F2. Results excluding immigrant samples

	Memory Test Score	Calculation Test Score	Time Orientation Test Score
Within 1 year of exposure	-0.301*	0.316	-0.191*
	(0.164)	(0.202)	(0.106)
Within 1 to 3 years of exposure	-0.651***	0.381	-0.405***
	(0.265)	(0.252)	(0.117)
More than 3 years of exposure	-0.107	0.361	-0.458**
	(0.248)	(0.250)	(0.176)
Individual FE	Y	Y	Y
Changwat (province) FE	Y	Y	Y
Year FE	Y	Y	Y
Interview month FE	Y	Y	Y
Interview day FE	Y	Y	Y
E_2 & E_3	Y	Y	Y
Covariates	Y	Y	Y
Observations	8014	5424	6615
R-squared	0.611	0.643	0.644

Notes: Standard errors clustered at the level of changwat (province) are in parentheses. This table reports standardized coefficients. FE indicates fixed effects. *** p<.01, ** p<.05, * p<.1
