# Supplementary material for: Flooding and Cognitive Health among Middle-Aged and Older Adults in Thailand: A Case Study of Resilient City Policy in Bangkok
Source: Ann Glob Health. 2025 Aug 19;91(1):49. doi: 10.5334/aogh.4740 (PMC12372663; doi:10.5334/aogh.4740)
Supplement: Supplementary Appendix F3. — Results excluding heavy flooding. [file agh-91-1-4740-s9.pdf]

F3. Results excluding heavy flooding

	Memory Test Score	Calculation Test Score	Time Orientation Test Score
Within 1 year of exposure	-0.288	0.196	-0.186
	(0.199)	(0.155)	(0.137)
Within 1 to 3 years of exposure	-0.433**	0.262	-0.275**
	(0.186)	(0.251)	(0.120)
More than 3 years of exposure	-0.096	0.321	-0.425**
	(0.352)	(0.286)	(0.186)
Individual FE	Y	Y	Y
Changwat (province) FE	Y	Y	Y
Year FE	Y	Y	Y
Interview month FE	Y	Y	Y
Interview day FE	Y	Y	Y
E_2 & E_3	Y	Y	Y
Covariates	Y	Y	Y
Observations	8015	5425	6616
R-squared	0.613	0.642	0.644

Notes: Standard errors clustered at the level of changwat (province) are in parentheses. This table reports standardized coefficients. FE indicates fixed effects. *** p <.01, ** p <.05, * p <.1
